# Antibiofilm Properties of Interfacially Active Lipase Immobilized Porous Polycaprolactam Prepared by LB Technique

**DOI:** 10.1371/journal.pone.0096152

**Published:** 2014-05-05

**Authors:** Veluchamy Prabhawathi, Thulasinathan Boobalan, Ponnurengam Malliappan Sivakumar, Mukesh Doble

**Affiliations:** Department of Biotechnology, Indian Institute of Technology Madras, Chennai, India; New York University, United States of America

## Abstract

Porous biomaterial is the preferred implant due to the interconnectivity of the pores. Chances of infection due to biofilm are also high in these biomaterials because of the presence of pores. Although biofilm in implants contributes to 80% of human infections [1], there are no commercially available natural therapeutics against it. In the current study, glutaraldehyde cross linked lipase was transferred onto a activated porous polycaprolactam surface using Langmuir-Blodgett deposition technique, and its thermostability, slimicidal, antibacterial, biocompatibility and surface properties were studied. There was a 20% increase in the activity of the covalently crosslinked lipase when compared to its free form. This immobilized surface was thermostable and retained activity and stability until 100°C. There was a 2 and 7 times reduction in carbohydrate and 9 and 5 times reduction in biofilm protein of *Staphylococcus aureus* and *Escherichia coli* respectively on lipase immobilized polycaprolactam (LIP) when compared to uncoated polycaprolactam (UP). The number of live bacterial colonies on LIP was four times less than on UP. Lipase acted on the cell wall of the bacteria leading to its death, which was confirmed from AFM, fluorescence microscopic images and amount of lactate dehydrogenase released. LIP allowed proliferation of more than 90% of 3T3 cells indicating that it was biocompatible. The fact that LIP exhibits antimicrobial property at the air-water interface to hydrophobic as well as hydrophilic bacteria along with lack of cytotoxicity makes it an ideal biomaterial for biofilm prevention in implants.

## Introduction

Biofilm is a complex matrix consisting of live and dead bacterial cells, exopolysaccharides, proteins and carbohydrates on a material surface. Such a biofilm on medical implants is a serious problem in biomedical applications. Antimicrobial coating including antibiotics is one of the most commonly used approaches for preventing biofilm in implants. Vancomycin when successfully attached to titanium exhibits bactericidal property against *Staphylococcus aureus* (*S.aureus*) and *Staphylococcus epidermidis* [2]. Chalcone and ZnO when coated on cotton cloth prevent the formation of *Staphylococcus aureus*, *Escherichia coli* (*E.coli*) and *Pseudomonas aeruginosa* biofilm [3]. Lipase embedded polycaprolactam is coimpregnated with an antibiotic, gentamicin sulfate and coated on urinary catheters to exhibit antimicrobial properties against *E.coli*, *Pseudomonas aeruginosa*, and *S.aureus*. [4]. Penicillin and ampicillin are covalently attached to expanded polytetrafluoroethylene through a PEG-spacer to develop antimicrobial surface [2], [5]. 2-Methoxy-2′, 4′-dichloro chalcone when mixed with marine paint and coated on polycarbonate, glass fiber reinforced plastic and polymethylmethacrylate prevents the formation of *Vibrio natriegens* biofilm [6]. However, in the above cases, antimicrobial property is exhibited as long as the compound is present on the surface. Leaching out of the compound from the surface leads to loss of antimicrobial property and hence this strategy is not suitable for implants that need to stay in the body for longer periods of time. In addition, development of drug resistance by the biofilm forming microbes is another serious problem which strongly requires strategies that do not use antibiotics [7].

Subtilisin, an enzyme, when immobilized on polycaprolactam exhibits antimicrobial activity against both Gram positive as well as negative microbes [8]. Silver nanocomposites also exhibit such properties [9]. Enzymes including oxidoreductases, transferases, hydrolases, esterases, isomerases and lyases have been reported to exhibit antibacterial property [10]. However, their mode of action and their effects in most of the cases have not been elucidated fully [10]. Lipase, a hydrolytic enzyme, exhibits antimicrobial and antifouling properties [11], but its mechanism of action is not studied so far. Polycaprolactam is a polymer with six amide bonds which lie in the same direction, resembling natural polypeptide. It is a porous polymer and is used as a scaffold for biomedical applications [12].

The significance of porous biomaterial for the construction of implants is stated in a work by Doi et al [13]. Such a material helps in osseointegration by forming a direct interface between the implant and bone without the intervening soft tissues [14]. Such implants are also more prone to the growth and proliferation of microbes [15]. These implants, in addition to supporting a damaged biological structure, could be made antimicrobial by incorporating an antimicrobial agent and made biosorbable by using a biodegradable polymer, which would prevent the need for another surgery to remove the implant. Enzymes are immobilized on these porous surfaces and used as biologically functionalized surfaces in enzyme delivery, diagnostic assays and bioreactors [13].

Langmuir Blodgett Deposition is a useful technique to design thin solid films at the molecular level [16]. When films are deposited on a porous surface, the monolayer will bridge the voids, supported by a layer of water. When the water drains or dries, the film collapses [17]. So, coating of molecules is improper and non uniform on porous surfaces. Maintaining the activity of protein and enzyme in the Langmuir-Blodgett monolayer is one more disadvantage that has not been completely overcome.

In the present study, Layer by Layer formation of highly active and stable biocatalytic film of cross linked lipase on a glutaraldehyde activated porous polycaprolactam surface is demonstrated by combining the immobilization and the Langumir Blodgett (LB) deposition procedures. Although biocatalytic properties of LB assemblies prepared in different ways are studied on glass surfaces [18], literature of such deposition on porous polymer surface for biological applications is minimal. Also, the mechanism of action of LB immobilized lipase on bacteria and their biofilm is presented here, which has not been reported anywhere.

## Experimental Section

### Materials

Purified lipase (EC 3.1.1.3), type VII from *Candida rugosa* (*C. rugosa*) (40 U of activity per mg of lipase, estimated by p-nitrophenyl palmitate assay), was purchased from Sigma (St. Louis, USA) and polycaprolactam from Marine industrial polymers, Chennai, India. Solvents used in the experiments were of HPLC grade (SRL, India). All the chemicals used for the biological studies were from Himedia (India). *S.aureus* NCIM 5021 and *E. coli* NCIM 293 were purchased from National Chemical Laboratory, Pune, India. They were stored in glycerol stock at −20°C and used when required.

### Organism hydrophobicity

The hydrophobicity of the microorganisms was determined by following a reported procedure [18]. The propensity of the organism to partition to hexadecane from aqueous phase was an indication of its hydrophobicity.

### MIC and Slimicidal activity of lipase

The minimal inhibitory concentration (MIC) [19], [20] of lipase and its slimicidal activity against *S.aureus* and *E.coli* were determined as per standard reported procedures [6].

### Preparation of preactivated polycaprolactam

Polycaprolactam was cut into 75×25 mm pieces and cleaned with 60% acetone solution followed by repeated rinsing with Millipore water of resistivity 18.2 MΩcm at 25°C. It was then incubated with 0.25% glutaraldehyde in 25 mM phosphate buffer at a pH of 4.5 (3.4 gm of monobasic sodium phosphate was dissolved in 1 liter of water and adjusted with 10N KOH to yield a 25 mM phosphate buffer of pH 4.5), under mild stirring for 15 h [21]. Then it was taken out, washed with phosphate buffer (25 mM, pH 4.5) followed by Millipore water (with resistivity of 18.2 MΩcm at 25°C) and was used immediately for coating.

### Crosslinking of lipase

Lipase from *Candida rugosa* with a molecular weight of 120,000 and isoelectric pH of 4.5 was cross linked with 0.25% of glutaraldehyde [22].

### Monolayer preparation and transfer

The preparation, characterization and deposition of monolayer of lipase onto pre-activated polycaprolactam were performed with a computerized, Teflon-bar-barrier type LB trough (Model No. LB-2007DC, Apex Instruments Co., India). The trough width and length were 40 and 21.5 cm respectively. Triple distilled and deionized Milli-Q water (Millipore model) which had a pH and resistivity of 6.8 and 18.2 MΩ cm respectively was used as the subphase. The surface pressure was measured using a Wilhelmy balance with an accuracy of ±0.01 mN/m. The experiments were performed at a pH of 4.5 (25 mM of phosphate buffer) and 25°C.

The cross linked lipase was used to form a monolayer on the water surface and this layer was transferred onto the preactivated polycaprolactam which was previously immersed in the subphase to prepare lipase immobilized polycaprolactam (LIP). The transfer was carried out at an evaporation time of 10 min, compression speed of 5 mm/min, dipping and lifting speed of 5 mm/min and 30 mins of drying time after each layer of coating. Lipase which was not cross linked with glutaraldehyde was deposited on non activated polycaprolactam surface using the same LB technique with same conditions that were used to prepare LIP and this surface was named as lipase coated polycaprolactam (LCP). Comparison of the performance of LIP and LCP was carried out to ascertain the advantages of the immobilization and coating strategy of the former method.

In each case, immediately after applying the enzyme as a thin LB layer, the substrate was placed in a desiccator maintained at 25°C for 24 h and its activity was determined as described below.

### Lipase activity

The lipase activity was determined by following a reported procedure using p-nitrophenol palmitate as the standard by estimating the release of p-nitrophenol from it [23]. One unit of lipase activity (U) was defined as the amount of lipase needed to liberate 1 µmol of p-nitrophenol per minute. The total activity of the immobilized lipase is the difference between the activity of the initial lipase used for immobilization and activity of unimmobilized (free) lipase. The activity of lipase immobilized on LIP was directly estimated with the substrate, which gave an indication if the immobilized lipase retained its activity or not. Residual activity is determined (as mentioned above) by converting the activity of enzyme present on LIP into percentage.

### Characterization of lipase immobilized surface

LIP was placed in a vial containing 25 mM of phosphate buffer, and incubated at different temperatures (25, 40, 60, 80 and 100°C) for one hour. The FTIR spectrum were recorded in the frequency range of 400–4000 cm^−1^ by ATR mode using Perkin Elmer PE 1600 FTIR spectrometer.

The activity of LIP of size 1×1 cm was measured at different pH values (ranging from 5 to 10 in steps) of one and at different temperatures, (ranging from 30 to 100°C in steps of 10°C). The activity of the free lipase in solution was also measured at the above mentioned pH and temperature conditions. The free lipase and LIP were stored at 4°C and the activity of the enzyme was monitored once in every 5 days for a total period of 40 days to check the storage stability.

The surface topography and the roughness of the UP and LIP surfaces mounted on a piezo electric scanner were measured with a nanoscope III atomic force microscope (3100 Controller, di Digital Instruments, Veeco, California) equipped with an ADCS control, in contact mode with a silicon nitride cantilever.

Sessile drop technique [19] was employed to measure the contact angle of these polymers with a Goniometer (Kruss, Germany) using Millipore grade distilled water.

### Characterization of biofilm

*E.coli* and *S.aureus* were grown on UP and LIP (of size 1×1 cm) in nutrient broth for 24 hours. Then the viable colonies in the biofilm formed on these surfaces were estimated according to a reported procedure [6]. Protein and carbohydrate in the biofilm were estimated as per Lowry's method using crystalline bovine serum albumin as the standard and phenol sulphuric acid method using glucose as the standard respectively [6].

Zeta potential of both the microbes grown on these films was estimated according to a reported method, with a few modifications [24]. 1×10^7^ number of microbes along with 1×1 cm of UP or LIP were cultured in a 25 ml nutrient broth for 24 hours. Then the polymer was removed, sonicated in 1 ml of nutrient broth and the OD values were adjusted to 0.1 at 600 nm using an UV spectrophotometer (Perkin Elmer, Lambda 35, Shelton, USA). The measurements were made with a Microtrac inc. nanotrac particle analyzer (Model: zetatrac; serial number MW12031907-U2839Z, USA). Experiments were carried out in triplicate. The motility of the microbes attached on the surfaces was measured using the same instrument.

The biofilm grown on both the surfaces were fixed with glutaraldehyde (0.1% in 25 mM phosphate buffer (14.55 gm of KH_2_PO_4_ was dissolved in 100 ml of water and adjusted with 0.1 M NaOH to yield a 25 mM of phosphate buffer of pH 7) for an hour [6], washed twice with 25 mM of phosphate buffer at a pH of 7.0 and once with distilled water, dried overnight in a dessicator, coated with gold and were viewed under a Scanning Electron Microscope (Jeol JSM 5600 LSV model).

The live and dead cells present in the biofilm after 24 hr of incubation were observed using a mixture of two nucleic acid staining dyes namely, SYTO9 and propidium iodide (PI) (Baclight, Invitrogen, USA) [5]. The former stains all live cells green whereas the latter dye enters only the dead cells (i.e. membrane damaged cells) and fluoresces red. The biofilm was grown on the two polymer surfaces and washed with distilled water. Then 20 µl of the dye mixture was placed on them and incubated in dark for 10 min. Excess dye was washed and these films were viewed under a fluorescence microscope (Leica DM5000, Germany) with a blue filter at a wavelength range of 420 to 495 nm [25].

Lactate dehydrogenase (LDH) activity was determined in the culture supernatants using NADH as the substrate by following a standard methodology [26].

### Cytotoxicity of the surfaces

3T3 cells (1×10^5^ cells/ml) were cultured in Dulbecco's Modified Eagle Medium and seeded in a 24 well plate followed by incubation in 5% of CO_2_ at 37°C until they attained confluence [9]. The UV sterilized polymers were washed with PBS of pH 7.0 and transferred into the wells of the plate and incubated for 48 h. The supernatant was discarded and 200 µL of MTT (3-(4,5-Dimethylthiazol-2-Yl)-2,5-Diphenyltetrazolium Bromide) solution was added to each well and incubated for 4 h. The supernatant was again discarded and 150 µL of dimethyl sulfoxide was added to each well, then the plate was covered with aluminium foil and left for an hour in a shaking incubator at 50 rpm. The absorbance was measured at 550 nm in an ultraviolet spectrophotometer (Perkin-Elmer, Lambda 35, Shelton, CT).

### Statistical analysis

All the analysis were repeated thrice on three independent samples (UP, LIP and free lipase) and were reported as mean ± standard errors (SE) of three samples. One way ANOVA, power analysis and two sample t-test were performed using MiniTab Ver 14.0 (MiniTab inc, USA). A p value<0.05 was considered to be statistically significant.

## Results and Discussion

**Figure 1:**
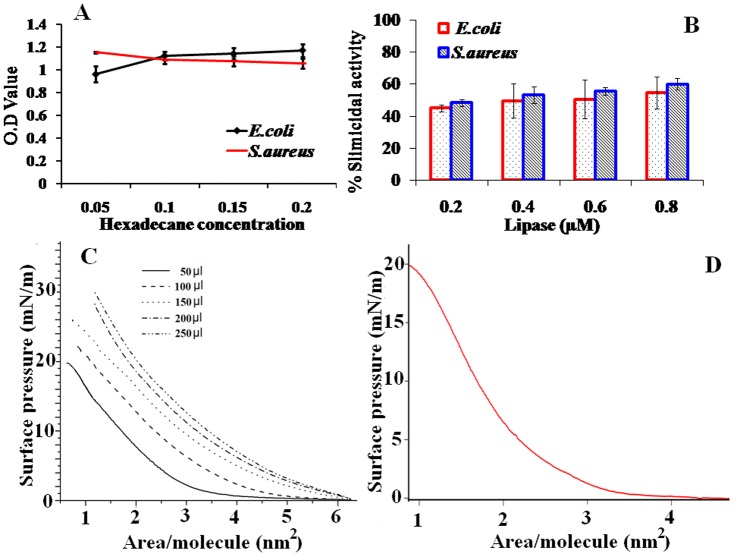
Hydrophobicity of microorganisms measured at varying volumes of hexadecane (BATH test) (A). Slimicidal activity of various concentrations of lipase against *E.coli* and *S.aureus* (B). Surface pressure-area isotherm (at a pH of 4.5 and 25°C) while preparing Lipase immobilized polycaprolactam (LIP) with different lipase amounts and 0.25% glutaraldehyde (C). Surface pressure-area isotherm of Lipase coated polycaprolactam (LCP) after optimization (D).

Surface property of the microbe plays a significant role in its adherence to a biomaterial. Decrease in OD with increase in hexadecane concentration (Figure 1-A) indicates that *E.coli* is hydrophobic while reverse trend observed in the case of *S.aureus* indicates that this organism is relatively hydrophilic. *E.coli* contains certain hydrophobic proteins in their cell wall which make them lipophilic [27]. *S.aureus* remains hydrophilic during its exponential phase [28], and generally hydrophobic in the stationary phase but loss of surface proteins, presence of capsules or the production of slime makes it hydrophilic. Microbe with greater surface hydrophobicity adheres to hydrophobic surfaces more than hydrophilic ones. So, it is highly challenging to remove a hydrophobic microbe such as *E.coli.* The effect of lipase on a hydrophilic and a hydrophobic microbe is studied here.

The MIC of lipase against *S.aureus* and *E.coli* are 0.1 and 0.05 µM respectively. In a biofilm forming microbe, exopolysaccharide (EPS) plays an important role in bacterial retention and transport. 100 µL of lipase at a concentration of 1 mM exhibits 65 and 50% slimicidal activity against *S.aureus* and *E.coli* respectively (Figure 1-B). Even the lowest concentration of lipase (0.2 µM) exhibits slimicidal activity (of 50 and 45% respectively) against both these organisms. Since lipase, an esterase, is a hydrolyzing enzyme, it is able to act on the EPS produced by the organisms [29], by degrading the high molecular weight lipid and protein components [30] of the biofilm. This preliminary experiment indicates that lipase can disturb the preformed biofilm that is attached to the surface of a biomaterial.

### Covalent linkage of lipase on polycaprolactam

The effect of glutaraldehyde concentration (from 0.010 to 1.0%) and reaction time (1–24 hr) on the activity of the cross linked lipase are studied (Table 1) and the condition which gives the highest activity is selected for preparing the Langmuir Blodgett thin film. The highest activity (of 4500 units) is observed at a glutaraldehyde concentration of 0.25% in 10 hrs. Comparison of surface pressure-area isotherms of different lipase (crosslinked) concentrations at the air/water interface with a glutaraldehyde concentration of 0.25% is shown in figure 1-C. Above the glutaraldehyde concentration of 0.25%, the lipase activity decreases (Table 1), since extensive cross linking results in distortion of the enzyme structure [31]. The cross linking experiments are performed at a pH of 4.5 which is the isoelectric point of *Candida rugosa* lipase. When this cross linked lipase is deposited using LB, the surface pressure starts to increase when the area/molecule is about 3.3 nm^2^. The monolayer passes from the gas to liquid state when the area/molecule is between 3.3 and 3.0 nm^2^ and remains in the liquid phase until the surface pressure reaches approximately 18 mN/m. There is a small plateau between 18 to 20 mN/m, due to the partial squeeze-out of the enzyme, preceding the full collapse [32]. This phenomena is due to the high pressure, resulting in desorption of the hydrophobic moieties of lipase from the air/water interface [32]. It is observed that no transfers can be done at surface pressures above 20 mN/m, possibly due to the fact that the crosslinked lipase undergoes a conformational change [33]. At this surface pressure, the film will be more compact. At higher concentration of lipase (100 to 250 µl of lipase), the isotherm goes to liquid state without the formation of gaseous state, which makes the formation of monolayer impossible (Figure 1-C). A sigmoidal type of behaviour is observed during the deposition when the process is operated at the isoelectric point of the enzyme and at a lipase concentration of 50 µl. This aids in uniform monolayer coating on the porous surface [33]. In this case, sigmoidal graph is observed at a lipase concentration of 50 µl. Compression isotherm of unimmobilized lipase on polycaprolactam surface, under similar experimental conditions is shown in figure 1D.

**Table 1:** Effect of glutaraldehyde concentration and time on lipase activity (4000 Units of lipase was used for crosslinking) at a pH of 4.5 (3.4 gm of monobasic sodium phosphate was dissolved in 1 liter of water and adjusted with 10 N (KOH) to yield a 25 mM phosphate buffer of pH 4.5).

Glutaraldehyde concentration (%)	Time taken for cross linking (h)	Lipase activity after crosslinking (Units)
0	0	4000±28 (Lipase activity)
0.016	23	3985±63
0.031	15	4100±32
0.0625	12	4125±24
0.25	10	4500±40
0.5	7	3685±25
1	4	2500±26

Here, poor adhesion is expected between the lipase monolayer and the hydrophobic porous surface of the polycaprolactam since they are bound by weak van der waals forces [34]. Whereas, interaction through glutaraldehyde molecules in the LIP leads to stable covalently cross linked layer of enzyme [34]. One of the serious problems of LB based material is the low mechanical stability of the multilayer films due to the lateral mobility of the molecules, especially in the presence of water [35].

### Surface analysis of LIP and LCP

**Figure 2:**
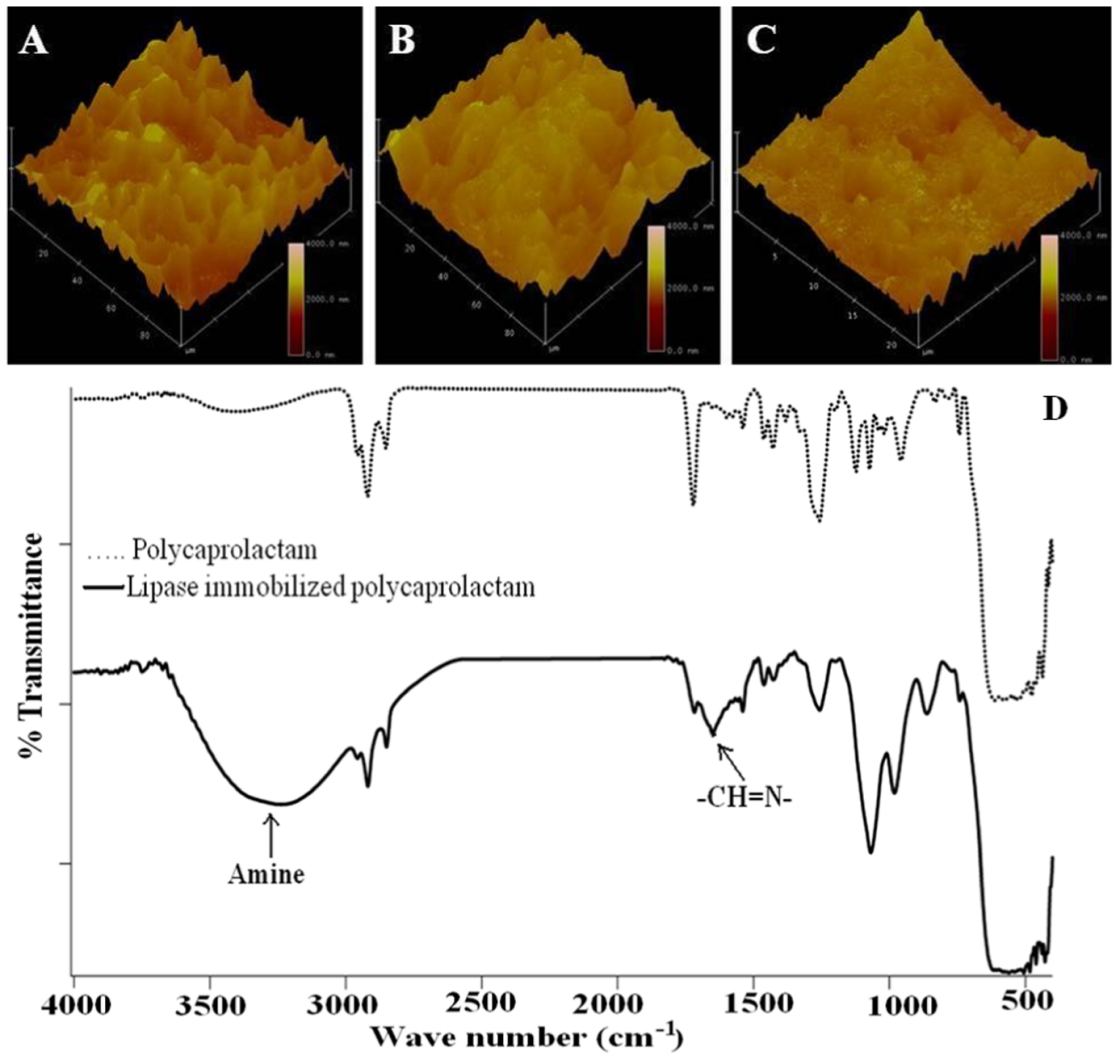
AFM images of surfaces of (A) polycaprolactam (UP), (B) lipase coated polycaprolactam (LCP) after buffer wash (C) lipase immobilized polycaprolactam (LIP) after buffer wash. (D) FTIR spectra of UP and LIP.

The AFM images of the UP, LCP and LIP surfaces after a buffer wash are shown in Figures 2 (A–C). The root mean square roughness of UP, LCP and LIP are 25±4 µm, 18±5 µm and 5±1 µm respectively.

Roughness is a very critical parameter while designing surfaces that will prevent cell attachment since it has been observed that nanometer scale roughness enhances the cell-surface interaction even if the surface is chemically uniform [36]. Moreover, porous surface such as polycaprolactam is more favorable for attachment and colonization of microorganisms than solid ones. Chemical heterogeneities on the surface create localized sites with high interfacial energy which leads to the deposition of the microorganism. In the present study, since LCP has rougher surface than LIP, there is a greater chance of adhesion of the microorganism on the former than on the latter, once again emphasizing the advantages of this method of immobilization over the other conventional LB deposition techniques.

Examination of infrared spectra of UP and LIP (Figure 2D) reveals a characteristic broad peak at 1646 cm^−1^ in the latter which is due to the bond between the carbonyl group of glutaraldehyde and imine (C = N stretching vibration) group of lipase as well as polycaprolactam. In addition, the intensity of the peaks due to amine (at 3189 cm^−1^) and due to imine (1653 and 1610 cm^−1^) increases in LIP as a result of the immobilization of the lipase to the polymer [6].

### Comparison of activities of LIP, and LCP and free lipase

The lipase on LIP was 50% more active than on the LCP surface (Table 2). This could not have been possible if conventional protein deposition techniques were adopted [37]. The detachment of lipase molecules from the LIP surface after repeated buffer wash (Table 2) was insignificant. Glutaraldehyde cross linking lead to substantial improvement in the stability of the lipase, whereas, its activity on LCP surface dropped by 50% after three buffer washes. Depositing the lipase without using any cross linking agent did not lead to a stable surface. So, further biological studies reported here were carried out only with LIP.

**Table 2:** Effect of different coating methods on the activity of lipase (10 µl of lipase with 10 units of activity was deposited on 75×25 mm polycaprolactam surface) (*p<0.05).

Polymer	Condition	Lipase activity on 1×1 cm piece (Units)
**LCP**	Before buffer wash	0.4385±0.011
	After buffer wash	0.2542±0.008*
**LIP**	Before buffer wash	0.7863±0.003
	After buffer wash	0.7860±0.001

Each 1×1 cm LCP and LIP was washed with 1 ml of 25 mM phosphate buffer (3.4 gm of monobasic sodium phosphate was dissolved in 1 liter of water and adjusted with 10 N (KOH) to yield a 25 mM phosphate buffer of pH 4.5).

The hydrophilic or hydrophobic characteristics of the surface influences bacterial adhesion and formation of biofilm [38], [6]. Hydrophobic bacteria prefer to adhere on hydrophobic surfaces [39] and vice versa. The contact angle of LIP (74.4±1.33°) is lower than that of UP (79.3±1.75°) indicating that the latter was relatively more hydrophobic than the former (p<0.05).

### Thermal and pH stability of LIP

Immobilizing an enzyme in solid matrices and at the same time preserving its catalytic activity is a challenge. Maximum lipase activity for both the free as well as the LIP were observed at a pH of 7 (Figure S1-A). Immobilization of lipase on the polymer increased its activity by 20%, which indicated that the catalytic site of the lipase was fully exposed making it available for hydrolytic function [40].Uniform arrangement of immobilized enzyme on LB coated polymer surface could prevent its aggregation, while free enzyme might aggregate and reduce its activity.

**Figure 3:**
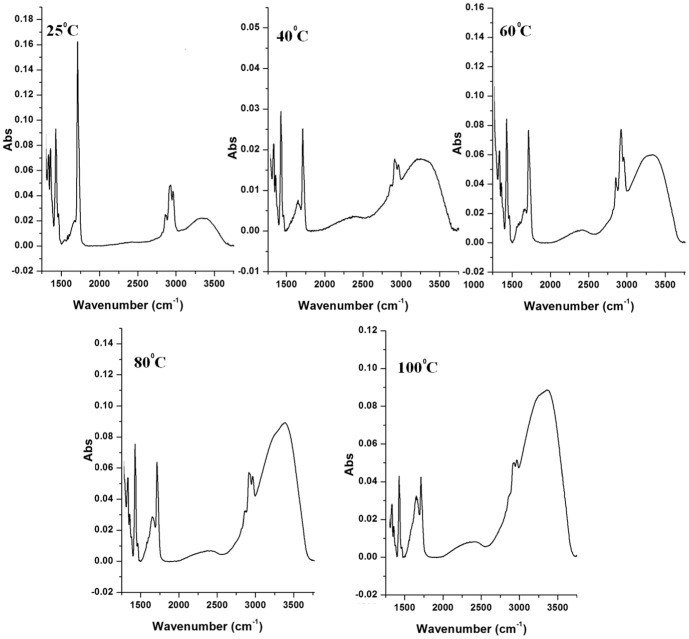
Changes in FTIR spectra of lipase immobilized on polycaprolactam (LIP) as a function of temperature.

The FTIR spectra (Figure 2D) of LIP indicates the presence of -C = N- group at 1599 cm^−1^ and a broader amine peak at 3000 to 3500 cm^−1^ confirming the immobilization of lipase on polycaprolactam. The FTIR absorption spectra of LIP as a function of temperature are shown in Figure 3. There are no changes in the intensity of the peaks in the region that characterize the C = N imino group (1590–1690 cm^−1^). This indicates that the enzyme is not thermally degraded. A small peak in the region of 1580–1690 cm^−1^ confirms that the covalent linkage between the enzyme and polycaprolactam also remains stable. Main bands in the region of 1250–1340 cm^−1^, 1280–1350 cm^−1^ and 1310–1360 cm^−1^ confirm the presence of aromatic primary, secondary and tertiary amines respectively. The absorbance value keeps increasing with increase in temperature, probably due to the swelling of the polymer which probably exposes the lipase that is deep inside the pores.

The activity of both the free as well as LIP reached a maximum at 60°C (Figure S1-B). The activity of the latter was 50% more than that of the free form (p<0.01). As the temperature was increased above this value, hydrolytic activity drastically decreased for the free lipase (denaturation) whereas the activity drop for the LIP was not so drastic and remained active until 100°C. It was reported that multipoint covalent immobilization of a macromolecule stabilized it making it stable towards harsh conditions including high temperatures and extreme pH values [41]. Also immobilizing the lipase at an interface would prevent its refolding and aggregation. It was reported that the secondary structure of the protein in a LB film was slightly affected only at 200°C, while in solution the same protein denatured at 60°C [42].

The possible reasons for the enhanced activity observed when coated on a surface using LB technique were the increased ordering of lipase when thin films were formed, making the protein confirmation more compact and thereby pressing its lid that was covering the active site to open. It was known from crystallographic studies that the activation of lipase involved the opening up of the lid that was covering its active site [43].

### Storage stability of LIP

LIP and UP retained 70 and 20% of their original activity after 40 days when stored at a pH of 7 and temperature of 4°C (Figure S1-C). Multipoint covalent immobilization was said to improve the stability by preventing aggregation and proteolysis. Similar observations were made while monitoring the storage stability of immobilized subtilisin [8]. LIP retained 65% of its activity at the end of one year. Retaining considerable activity and stability even after one year of storage indicated that LIP provided significant advantage over the free lipase. Except for this study on residual activity, no other experiments were performed to test the stability of lipase, which would be performed as a next level of study.

The activity retained by LIP will also be relevant on the duration of stay of this material in the body. For short duration implants, the activity lost is only marginal. Although the short term behavior of this surface appears promising, longer term studies extending to months need to be performed depending upon the application.

### Characterization of Biofilm

**Figure 4:**
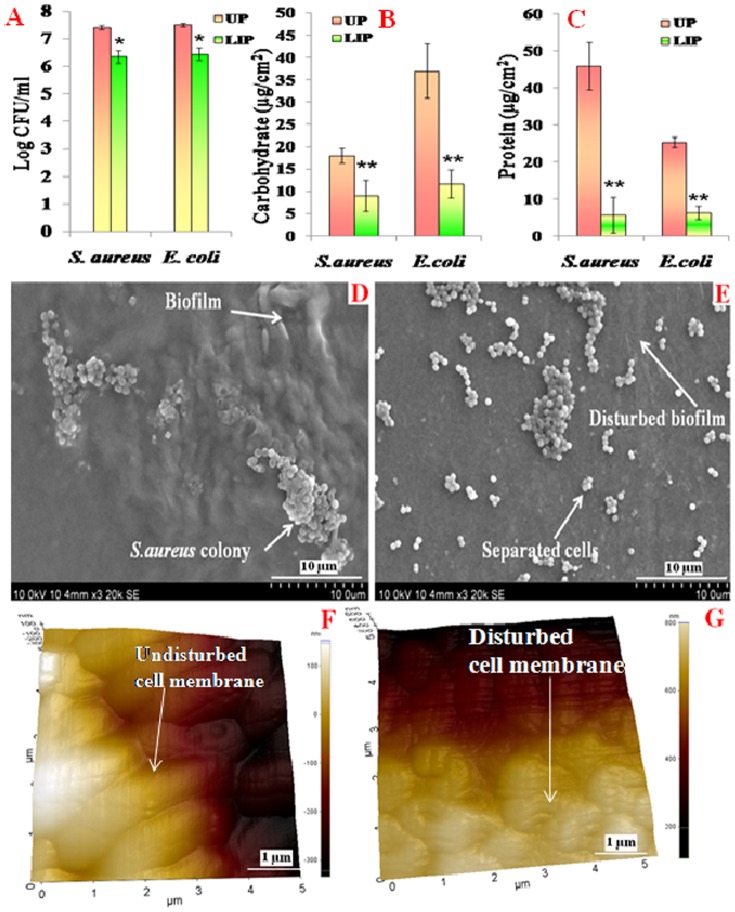
Biofilm formed on UP and LIP surface (A) CFU, (B) carbohydrate and (C) protein (**p<0.001; *p<0.01). SEM images of (D) *S.aureus* adhered on UP and (E) LIP surfaces after 24 hr. AFM images of *E.coli* on (F) UP and (G) LIP.

Biofilm is a complex structure and any ideal antifoulant must exhibit activity against live and dead cells, glycocalyx, exopolysaccharides, proteins and carbohydrates [44]. The hydrophobic nature of *E.coli* resulted in more adhesion of it on polycaprolactam surface when compared to the hydrophilic *S.aureus* (Figure 4-A). About ten times reduction in CFU of both the organisms were observed on LIP when compared to the attachment on UP (Figure 4-A), indicating the antibacterial nature of lipase. In addition the former surface was smoother than the latter which also decreased bacterial attachment.

*E.coli* appeared to produce more carbohydrate than *S.aureus* (Figure 4-B). 2 and 3.5 times reduction in carbohydrate were observed on LIP when compared to it on UP when they were exposed to *S.aureus* and *E.coli* respectively. Although the total amount of polysaccharide present in the biofilm on the LIP is lesser than that on the UP, one cannot conclude that polysaccharide produced by the organisms has reduced due to the enzyme action in the former, because the number of colonies has decreased due to the bactericidal activity of the lipase. So, we cannot say that the EPS per live colony is decreased due to lipase

Biofilm protein was higher on the UP surface that was exposed to *S.aureus* when compared to that exposed to *E.coli* (Figure 4-C). It was less on LIP than on UP surface, probably because of the antibacterial activity of the lipase as well as relatively hydrophilic nature of the former than the latter surface. These proteins preferentially attach on hydrophobic surfaces [44]. Increase in the hydrophilicity of the surface will decrease the attachment of bacteria which may lead to reduction in the biofilm [45]. Imparting hydrophilic characteristic to the polymer is one method of preventing biofilm [6], while other techniques include imparting antibacterial properties, for example by using subtilisin [8] or protein stabilized silver nanoparticles [46].

Lipolytic enzyme catalyzes reactions on a lipid substrate including phospholipids and other hydrophobic molecules, to hydrolyze or esterify a bond. Here lipase exhibits antibacterial activity by acting both on the lipopolysaccharide of Gram negative cell wall as well as the esters of exopolysaccharide present in the biofilm. It is reported that ctivity of lipase increases when it is placed at the hydrophilic/hydrophobic interface [47]. Lipase exists in two main forms, open and closed [48]. In aqueous medium, the lid or flap remains closed making it inactive, while it remains open in the presence of natural substrates including oil, converting it to an active form, known as interfacial activation. Immobilization on a support would give it a dispersed open form, cleaving this lid [49]. Moreover, by changing the support morphology and hydrophobicity, it is possible to yield an open form which is highly active in any substrate. Since biofilm is formed at the interface, employing an interfacial enzyme such as lipase fulfills the requirement of the prevention of the former.

Zeta potential is an indication of the attractive forces that play between the bacteria and the surface, and it can explain why certain bacterial cells are tough to be eliminated from biomaterial surface when compared to others. The zeta potential of *E.coli* and *Staphylococcus aureus* on LIP were −19.36±0.55 mV and −24.39±−2.65 mV respectively (Table 3). Out of these two microbes, the former has the highest zeta potential on UP (−2.65±−0.96 mV), which leads to strong adhesion on the solid surface, making it highly challenging to be eradicated, when compared to *S.aureus*. Negative zeta potential of microbial cells on LIP when compared to that on UP indicates that repulsive force was high between the microbe and the former than the latter polymer surface leading to reduction in their attachment [50]. Reduction in zeta potential suggests charge neutralization, leading to less microbial adhesion and formation of biofilm.

**Table 3:** Zeta potential and mobility of bacteria on UP and LIP surfaces.

	Zeta potential (mV)		Mobility (µm/s/V/cm)	
	Bacteria on UP	Bacteria on LIP	Bacteria on UP	Bacteria on LIP
***E.coli***	−2.86±0.96	−19.36±0.55	4.11	2.11
***S.aureus***	−8.65±0.63	−24.39±2.65	2.89	0.96

Motile populations, such as swarming bacteria, can rapidly reach niches, which they can colonize [51]. In the present study, the motility of *E.coli* on UP was 4.11 µm/s/V/cm; which decreased to 2.11 on the LIP surface (Table 3). The motility of *S.aureus* on the UP and LIP surfaces were 2.89 and 0.96 respectively. These results also correlated with the reduced amount of biofilm formed on LIP when compared to that on UP. The motility of *S.aureus* is less than that of *E.coli* indicating the intrinsic biofilm forming ability of the former than that of the later.

### Microscopic analysis

The SEM micrographs showed *S.aureus* to be well spread out on the uncoated polymer (Figure 4D). Whereas the LIP surface was predominantly devoid of any cells (Figure 4E) corroborating the CFU measurements.

The AFM image (Figure 4F) of *E.coli* on UP showed clear outer membrane which was reasonably structured. The surface of the cell was without any pores or undulations. The average length and width of the cells were 2.3±0.8 µm and 1.8±0.23 µm respectively, which matched with those reported in the literature [52], [53]. It was clear that the *E.coli* cells had not undergone any structural changes over a period of 24 hours, namely maintaining a typical rod shaped morphology (Figure 4F). Whereas, visible damage in its outer membrane was evident on LIP surface (Figure 4G). Vesicles might form in areas where linkage of the outer membrane and peptidoglycan was weakened [54]. Also, a pronounced collapse in the mid region of the bacterial envelop was seen here (Figure 4G). This could lead to leakage of the cytoplasm. Bacterial cells exposed to LIP released nine times more LDH than those exposed to UP confirming the action of lipase (in LIP) on the cell wall leading to the leakage of its contents.

The root mean square roughness of bacteria grown on UP and LIP surfaces as estimated with AFM were 74.47±13.32 and 27.74 0±3.87 µm respectively. More bacterial growth on the former polymer than on the latter might have lead to the observed difference.

**Figure 5:**
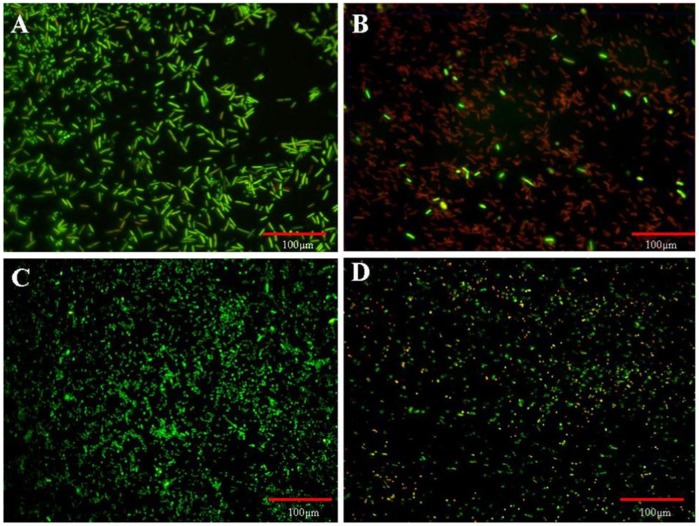
Fluorescence microscopic images of *E.coli* and *S.aureus* formed on UP (A & C) and LIP (B & D) surfaces respectively after 24 h of incubation. Green dots represent live and red colour represents dead cells.

The fluorescence image of UP surface (Figure 5A) showed more green than red spots indicating more number of live *E.coli* cells than dead ones. The LIP surface showed red spots indicating dead (*E.coli* membrane damaged) cells (Figure 5B). Similarly, more live *S.aureus* cells could be seen on UP than on LIP surface (Figure 5C & 5D). This once again confirmed that lipase acted on the microorganism by damaging the cell wall.

### Cytotoxicity of polycaprolactam

**Figure 6:**
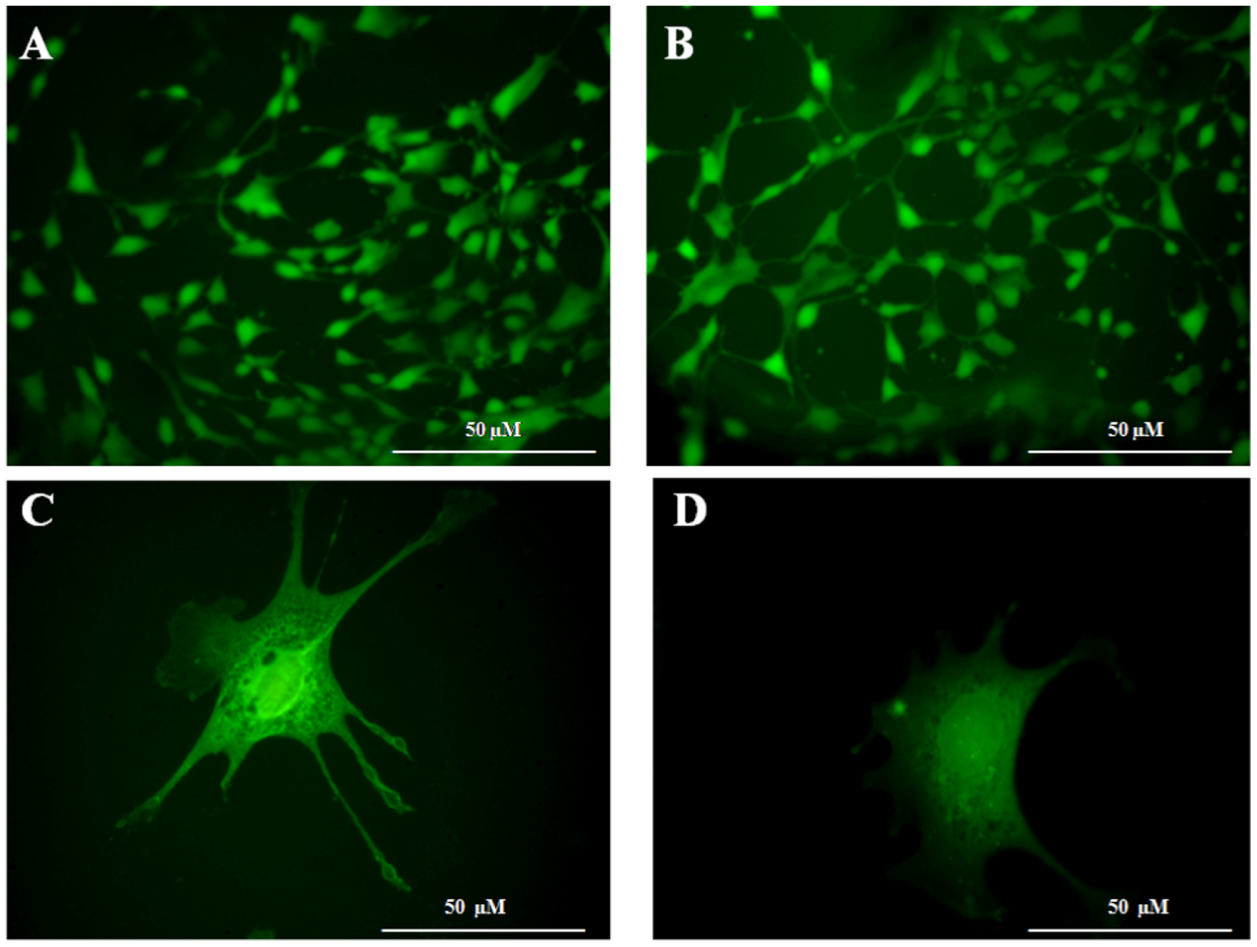
Micrographs showing 3T3 cells on the UP surface (A & C) and LIP surface (B & D).

Figures 6A & 6B show uniform growth of 3T3 cells over the LIP and UP surfaces. The morphology of the adipocytes on them appeared similar. Figures 6C and 6D indicated that there was very good proliferation and spreading of these cells on these polymers. After 48 hours of incubation, viable 3T3 cells on UP and LIP were 97.4 and 98.6% respectively, indicating that lipase present on the polymer surface did not impart any additional toxicity to the adipocytes. These results indicate that the modified polymer was biocompatible and could be used for implant applications.

### Long term antimicrobial effect of LIP on microbes

**Figure 7:**
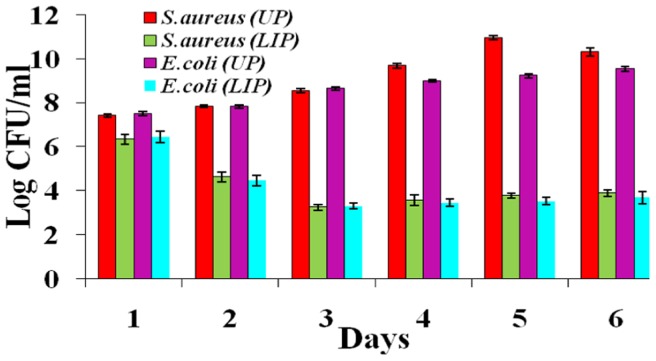
Long term antimicrobial effect of LIP and UP on *S.aureus* and *E.coli*.

Long term effect of LIP on *S.aureus* and *E.coli* was studied. Live colonies of microbes on UP kept increasing from 7 to 10 log CFU as the number of days of incubation increased from 1 to 6, whereas it decreased from 6 to 4 log CFU in the case of LIP (Figure 7). The enzyme is relatively less active on the first day and increasing the incubation time leads to the swelling of the polycaprolactam surface leading to exposure of more lipase and its active sites exhibiting sustained increase in the antibacterial activity.

### Statistical analysis

The power was calculated for all the data and it lies between 0.805 and 0.891.

### Concluding remarks on the mode of action of lipase

One of the common problems faced during LB thin film deposition of protein on a surface is its solubility in water and lack of stability. If the deposition pH is different from its isoelectric pH then it can carry a net charge, thus making it partially soluble and hence allowing it to dissolve into the subphase [55]. Here, depositing lipase at its isoelectric point results in the formation of a thin film on the aqueous surface without loosing its activity and stability. The multipoint covalent immobilization of an enzyme inside a porous support may have several protective effects on the structure of the former [41]. When the enzyme is present inside the pore, it remains stable and active in harsh environmental conditions. Also, LB immobilization creates stable film on porous surfaces.

LIP surface is relatively more hydrophilic and smooth than the UP surface thereby preventing the attachment of hydrophobic organisms including *E.coli*. LIP exhibits slimicidal activity as evidenced by the reduction in the carbohydrate. It has been estimated that biofilm cells are up to 1,000 times more resistant to most of the antimicrobial agents than planktonic cells [56] and, 80% of all bacterial infections are biofilm related [56]. So the antibiofilm property of the lipase could help in preventing the formation of such a matrix.

Outer membrane of the Gram negative bacterial cell is a lipid bilayer that forms a continuous barrier around it. Presence of lipopolysaccharide layer (75% of the total membrane surface) prevents the permeabilization of antibacterial within the bacterial cell [57]. Lipases are esterases capable of hydrolyzing any ester bond. They act on the lipoprotein, lipopolysaccharide and phospholipids which surrounds the peptidoglycan layer leading to the hydrolysis of the lipid bilayer. The lipopolysaccharide complex is an endotoxin present on the outer membrane of the cell wall and this toxicity leads to a wide spectrum of nonspecific pathophysiological reactions including fever, changes in white blood cell counts, disseminated intravascular coagulation, hypotension, shock and death. When lipase acts on this lipid A, the chances of infection is minimized [58]. In most of the Gram positive bacteria, lipoteichoic acids are present and the lipid tail present here plays a major role in the bacterial attachment. There is a possibility for the lipase to act on this lipid tail thereby preventing its adherence to a surface.

Lack of stability of protein molecule remains a major problem in LB coatings and more so on porous surfaces. So, immobilizing the protein using the present methodology will help in designing stable, active as well as uniform coating. Moreover, this study will form a basis for immobilizing various types of biomolecules on a wide range of porous surfaces. The coating demonstrated here is also biocompatible which makes it an ideal technique for use in implants.

## Supporting Information

Figure S1 (A) Activity of free lipase and LIP as a function of pH (p**<0.01). (B) Activity of free lipase and LIP as a function of temperature (**p<0.01). (C) Storage (4°C) stability of free lipase and LIP (**p<0.01).
(TIF)
