# Supplementary figures and images for: Antibiofilm Properties of Interfacially Active Lipase Immobilized Porous Polycaprolactam Prepared by LB Technique
Source: PLoS One. 2014 May 5;9(5):e96152. doi: 10.1371/journal.pone.0096152 (PMC4010425; doi:10.1371/journal.pone.0096152)

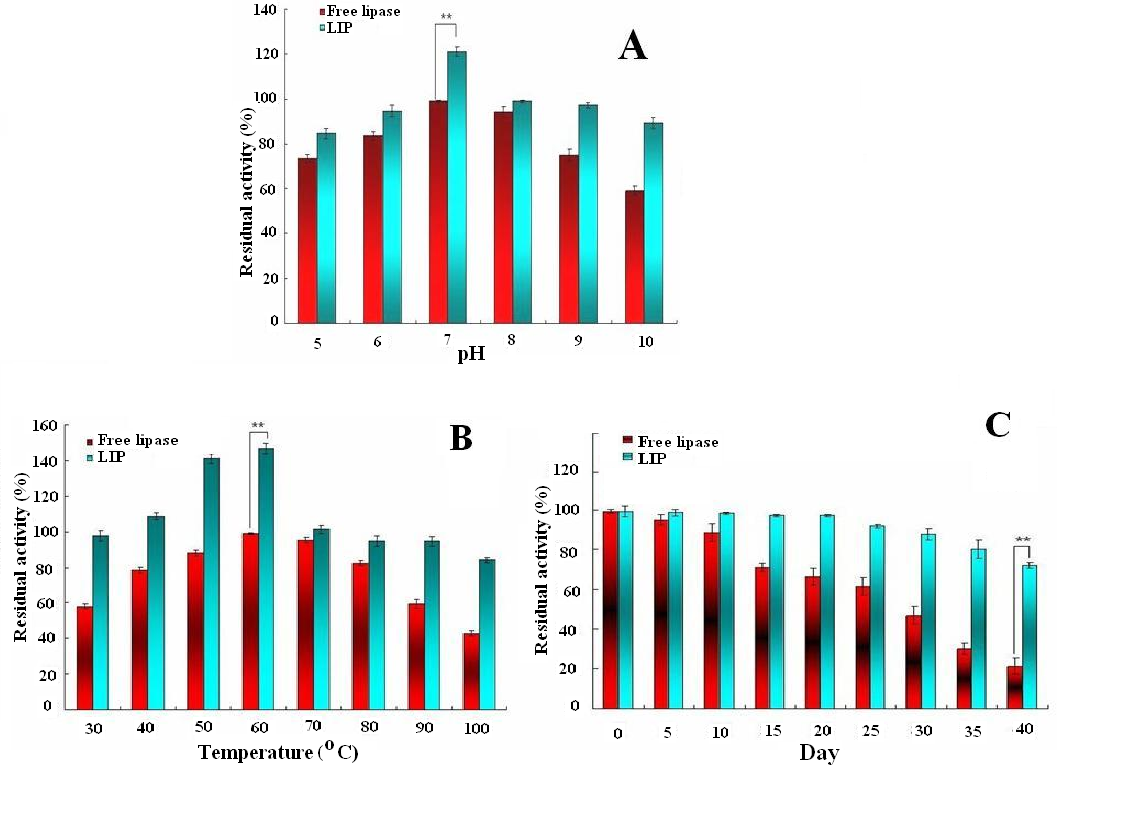

Supplement: Figure S1 — (A) Activity of free lipase and LIP as a function of pH (p**<0.01). (B) Activity of free lipase and LIP as a function of temperature (**p<0.01). (C) Storage (4°C) stability of free lipase and LIP (**p<0.01). (TIF) [file pone.0096152.s001.tif]
